# An Unexpected Cause of Severe Hypokalemia

**DOI:** 10.1155/2015/957583

**Published:** 2015-10-15

**Authors:** Fernando Caravaca-Fontan, Olga Martinez-Saez, Maria Delgado-Yague, Estefania Yerovi, Fernando Liaño

**Affiliations:** Department of Nephrology, Hospital Universitario Ramón y Cajal, Carretera de Colmenar Viejo, Km. 9100, 28034 Madrid, Spain

## Abstract

We describe an unusual case of severe hypokalemia with electrocardiographic changes, due to licorice consumption, in a 15-year-old female student with no previous medical history. Prompt replacement of potassium and cessation of licorice ingestion resulted in a favourable outcome. We also discuss the pathophysiology and diagnosis, emphasizing the importance of a detailed anamnesis to rule out an often forgotten cause of hypokalemia as the licorice poisoning.

## 1. Introduction

Hypokalemia is one of the most frequently encountered fluid and electrolyte abnormalities in clinical medicine. It can result from reduced potassium intake, transcellular potassium uptake, and extrarenal or renal potassium loss [1]. While underlying tubular disorders may be suspected in young patients, it is important to rule out secondary causes of hypokalemia, such as medications or herbal complexes [2]. Children and young adults tolerate hypokalemia better than elderly patients, although cardiac arrhythmia may occur in both cases. Prompt replacement of potassium to a safe level is mandatory.

## 2. Case Report

**Figure 1:**
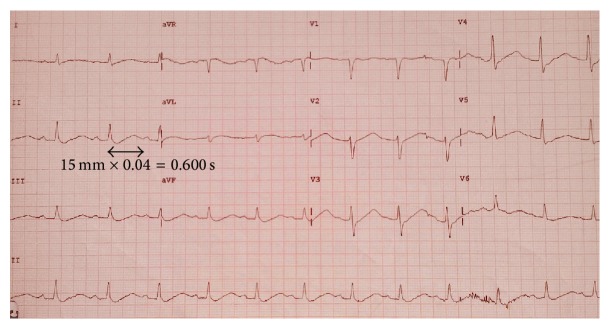
Electrocardiogram on admission shows sinus rhythm, mild increase in P waves, and mild depression of the ST segment. Note the prolonged QT interval of 600 milliseconds.

A 15-year-old female student with no relevant medical history was admitted to the emergency department with dizziness, nausea, and progressive weakness over the last 24 hours. She denied smoking, alcohol consumption, or diuretic use but recognized practicing aerobic exercise every day. Her familial medical history was unremarkable. She had not had dyspnoea, fever, vomiting, or diarrhea in the last days. Physical examination showed a blood pressure of 135/80 mmHg and heart rate of 75 beats per minute. Chest auscultation revealed regular heart sounds with no murmurs, and abdominal exam did not reveal tenderness, hepatomegaly, or masses. In the electrocardiogram sinus rhythm was described, with prolonged QT interval of 600 milliseconds (Figure 1).

The initial laboratory data showed severe hypokalemia of 1.8 mmol/L and metabolic alkalosis with pH 7.6, pCO2 48 mmHg, and HCO3 47 mmol/L. Serum creatinine, sodium, calcium, phosphate, magnesium, and uric acid were within the normal range. Mild elevation of creatine kinase of 321 U/L was observed. A urine sample analysis showed urinary sodium of 50 mmol/L and potassium of 65 mmol/L, with a transtubular potassium gradient of 16.

Over the next six days, the patient received high doses of intravenous and oral potassium chloride, with slow progressive correction, and further studies were performed in order to exclude other potential causes. Low plasma renin concentration [<0.3 ng/mL/h (normal values 0.3–7 ng/mL/h)], low plasma aldosterone [3.4 ng/dL (normal 5–40 ng/dL)], normal cortisol concentration [10.5 mcg/dL (normal 3–20 mcg/dL)], and normal adrenocorticotropic hormone levels [15 pg/mL (normal 5–45 pg/mL)] were found on specific hormonal analysis. Abdominal computed-tomography findings were unremarkable. Although a primary hereditary tubular disorder was the first clinical suspicion, after detailed anamnesis the patient confessed regular consumption of licorice roots over the last two weeks. The clinical course was favourable and the patient was discharged ten days after, with complete correction of these biochemical abnormalities, correction of electrocardiographic changes, and a clear advice of not taking licorice again.

## 3. Discussion

This case highlights the potential toxicity of licorice in the development of severe hypokalemia with electrocardiographic changes, which could eventually lead to fatal arrhythmia in certain cases. Licorice contains glycyrrhizin and has been used in traditional Asian medicine for its anti-inflammatory properties [3]. On the other hand, licorice is present in the western world as a natural sweetener for foods and candies, as well as in natural roots. It has been reported that the concentration of glycyrrhetinic acid (GA) in licorice roots can vary up to 20% depending on the extraction process [4].

**Figure 2:**
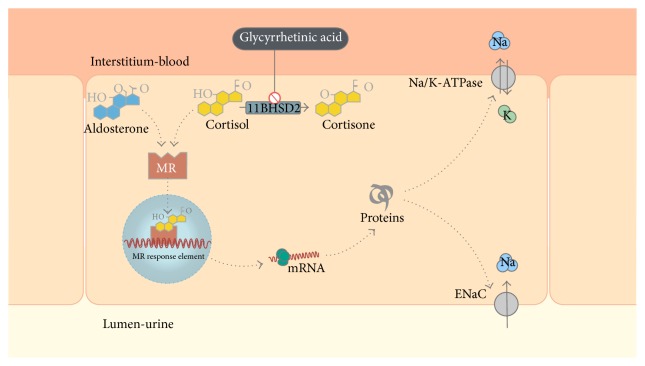
Aldosterone acts primarily in the distal nephron by diffusing into the tubular cell and attaching to specific mineralocorticoid receptor (MR). After that, the ligand-receptor complex migrates into the nucleus, where it interacts with specific sites and enhances messenger RNA (mRNA) and ribosomal RNA transcription. Aldosterone-induced proteins are synthesized, such as the apical epithelial sodium channel (ENaC) and basolateral Na/K-ATPase.* In vitro*, aldosterone and cortisol have similar affinity to the MR. The enzyme 11*β*-hydroxysteroid dehydrogenase type 2 (11BHSD2) converts cortisol to inactive cortisone, preventing cortisol from binding to the MR. Glycyrrhetinic acid inhibits 11BHSD2 leading to activation of MR by cortisol.

Under physiological conditions, aldosterone binds to the mineralocorticoid receptor (MR) in the principal cells of cortical collecting duct, resulting in signal transduction and subsequent expression of apical epithelial sodium channel and basolateral Na/K-ATPase. The net result is sodium reabsorption and potassium excretion [5]. The affinity of cortisol to MR is similar to that of aldosterone, but the presence of 11*β*-hydroxysteroid dehydrogenase type 2 (11BHSD2), an enzyme that converts cortisol to inactive cortisone, prevents cortisol from binding to the MR (Figure 2).

GA inhibits 11BHSD2 leading to activation of the MR by cortisol. Patients present with hypertension, hypokalemia, and metabolic alkalosis [6]. In most cases, potassium depletion develops slowly, and symptoms such as myalgia or cramps appear when serum potassium levels are severely low. Our patient needed high doses of potassium chloride, which may reflect a severe intracellular depletion of potassium.

Although hypokalemia is the most dangerous side effect of licorice consumption, the main adverse effect is hypertension. The USA Food and Drug Administration (FDA) advises avoiding eating large amounts of black licorice at one time [7]. An increase in extracellular volume and elevation of central systolic and diastolic blood pressure after two weeks of daily licorice consumption have been reported [8]. However, it has been suggested that individual factors may predispose to pseudoaldosteronism in certain patients, since some patients develop neither hypokalemia nor hypertension even after long time consuming licorice. In addition, some medications can interact with glycyrrhizin metabolism [3].

A precise diagnosis of hypokalemia secondary to licorice consumption requires a careful anamnesis, even though specific blood tests and urinalysis may give the definitive diagnosis. Urinalysis typically shows inappropriately elevated urinary potassium level, with a transtubular potassium gradient above 7, all of which with low plasma renin and aldosterone concentration [9].

A 24-hour urine collection may be necessary to rule out underlying tubular dysfunction. Decreased free urinary cortisone and an increased ratio of urinary free cortisol to urinary free cortisone are usually found in a 24-hour urine collection [9]. This may help make the diagnosis, but it is not required if a history of licorice ingestion has been obtained.

Other conditions to be included in the differential diagnosis of patients with hypokalemia, metabolic alkalosis, and hypertension are inherited disorders such as Liddle syndrome, apparent mineralocorticoid excess, and glucocorticoid-remediable aldosteronism [10]. These disorders are not correctable and patients usually need lifelong therapy with potassium supplements and potassium-sparing diuretics. In rare cases, incomplete phenotypes of tubular disorders become apparent after licorice ingestion.

In conclusion, it is important to keep in mind licorice abuse as a cause of symptomatic hypokalemia even in young patients. A careful anamnesis and a complete hormonal and urinary analysis are essential for diagnosis.
